# Comment on: Stress-induced hyperglycemia and expression of glucose cell transport genes in skeletal muscle of critically ill patients: a cross-sectional study

**DOI:** 10.20945/2359-4292-2025-0321

**Published:** 2025-10-08

**Authors:** Raahin Rahim, Hoorain Talpur, Fatima Khurshid

**Affiliations:** 1 Jinnah Sindh Medical University, Karachi, Pakistan; 2 Ibn-e-Sina University, Mirpurkhas, Pakistan; 3 Karachi Medical and Dental College, Karachi, Pakistan

Dear Editor,

We read with interest the paper by Bellaver and cols., entitled “Stress-induced hyperglycemia and expression of glucose cell transport genes in skeletal muscle of critically ill patients: a cross-sectional study” ^(1)^. This research provides useful information about metabolic changes in critical illness. The author presents a variety of perspectives with fairness and detail, maintaining a praiseworthy balance. We agree with the well-founded conclusion that *IRS1* was downregulated in patients with stress-induced hyperglycemia; however, some methodological weaknesses have been encountered, which deserve mention.

Initially, it is not established whether patients on corticosteroids or with liver dysfunction were excluded or controlled. Both are prevalent and independently affect glucose metabolism. Stress hyperglycemia was described by Marik and cols. ^(2)^ as a multifactorial physiologic response, partially mediated by hepatic gluconeogenesis and modulated by endogenous and exogenous corticosteroids. Without controlling for these variables, ascribing *IRS1* downregulation to stress-induced hyperglycemia alone is doubtful.

Secondly, the study is narrowly targeted on four genes (*IRS1, IRS2, SLC2A1*, and *SLC2A4*), when the intricacy of insulin signaling is well established. More global transcriptomic or pathway-based strategies would have given a much broader molecular understanding of alterations. Vorotnikov and cols. note that insulin resistance emerges from various interacting regulatory nodes beyond those discussed here, validating the necessity for OMICS-level analysis in such intricate pathophysiologic states ^(3)^.

Thirdly, due to the cross-sectional nature of the study, it remains uncertain if *IRS1* downregulation is a response that is acute or representative of pre-existing susceptibility. Rung and cols. recognized a variant close to the *IRS1* locus (rs2943641) that correlated with decreased *IRS1* expression and insulin resistance, independent of acute illness ^(4)^. This introduces the likelihood that there are genetic underpinnings to what is observed. Moreover, inflammation, characteristic of critical illness, was not controlled for. Pro-inflammatory signaling, particularly by the c-Jun N-terminal kinase (JNK) pathway, has been shown to suppress *IRS1* activation. Hommelberg and cols. illustrated that inhibition of JNK maintains *IRS1* function in the presence of inflammation and, by implication, could contribute to decreased *IRS1* expression in this group ^(5)^. Cumulatively, these variables defy crediting *IRS1* downregulation solely to hyperglycemia.

In conclusion, while this study addresses an important question, future research would benefit from broader molecular profiling, inclusion of inflammatory markers, and longitudinal designs to better clarify causality and gene regulation in stress hyperglycemia.

## Data Availability

datasets related to this article will be available upon request to the corresponding author.
